# Mortality Due to Aortic Dissection in Adults With Primary Hypertension: A Nationwide Analysis Over Two Decades

**DOI:** 10.1002/clc.70269

**Published:** 2026-02-05

**Authors:** Shahzaib Ahmed, Zain Ali Nadeem, Aimen Nadeem, Hamza Ashraf, Umar Akram, Eeman Ahmad, Shoaib Ahmad, Ibrahim Nagmeldin Hassan, Irfan Ullah, Raheel Ahmed, Anwar A. Chahal, Rui Bebiano Da Providencia E. Costa, Chadi Alraies

**Affiliations:** ^1^ Department of Medicine Fatima Memorial Hospital College of Medicine and Dentistry Lahore Pakistan; ^2^ Department of Medicine Allama Iqbal Medical College Lahore Pakistan; ^3^ Department of Medicine King Edward Medical University Lahore Pakistan; ^4^ St. Joseph Hospital and Medical Center Phoenix Arizona USA; ^5^ University of Khartoum, Faculty of Medicine Khartoum Sudan; ^6^ Department of Internal Medicine Khyber Teaching Hospital Peshawar Pakistan; ^7^ National Heart and Lung Institute, Imperial College London London UK; ^8^ Department of Cardiovascular Diseases Mayo Clinic Rochester Minnesota USA; ^9^ Center for Inherited Cardiovascular Diseases, WellSpan Health Lancaster Pennsylvania USA; ^10^ Barts Heart Centre, St Bartholomew's Hospital, Barts Health NHS Trust London UK; ^11^ NIHR Barts Biomedical Research Centre, William Harvey Research Institute Queen Mary University of London London UK; ^12^ Department of Cardiology University College London London UK; ^13^ Department of Cardiology Newham University Hospital, Barts Health NHS Trust London UK; ^14^ Institute of Health Informatics Research Univestity College London London UK; ^15^ Department of Cardiology Barts Heart Centre, Barts Health NHS Trust London UK; ^16^ Department of Cardiology Detroit Medical Center Detroit Michigan USA

**Keywords:** aortic dissection, hypertension, mortality

## Abstract

**Objective:**

Hypertension is a key risk factor for aortic dissection (AD). AD, if left untreated, carries significant mortality rates. Our aim is to analyse trends in mortality due to AD in adults with primary hypertension in the United States (US).

**Methods:**

We used the CDC WONDER database to extract mortality data for patients with primary hypertension who died due to AD. Age‐adjusted mortality rates (AAMRs) and crude mortality rates (CMRs) were extracted per 100 000 persons. Annual percentage changes (APCs) and average APCs (AAPCs) in AAMRs and CMRs were calculated using Joinpoint regression.

**Results:**

From 1999 to 2020, a total of 13 128 deaths due to AD were reported in patients with primary hypertension in the US. Males displayed a higher overall AAMR (0.3) than females (0.2) throughout the study period. Slight regional variations were observed, with the West showing the highest overall AAMR (0.4), followed by the Midwest (0.3), and the Northeast and South (0.2). In urban areas, AAMRs were higher than in rural areas until 2008. From 2009 to 2020, AAMRs remained stable in urban areas (0.3) but increased in rural areas from 2010 to 2020 (4.7). The highest state‐level AAMRs were observed in Hawaii, Oregon, and Oklahoma.

**Conclusion:**

Significant differences were observed in AAPCs compared to AD‐related mortality trends in the general population. Mortality trends revealed an initial decline followed by a gradual rise. Clinicians should focus on high‐risk groups and raise awareness about the disease in these populations.

## Graphical abstract

From 1999 to 2020, aortic dissection–related mortality in US adults with hypertension showed an initial rise, followed by stabilization. Mortality was highest in males, older adults, NH Black individuals, and the Western US, highlighting disparities and the need for improved hypertension control in high‐risk groups.

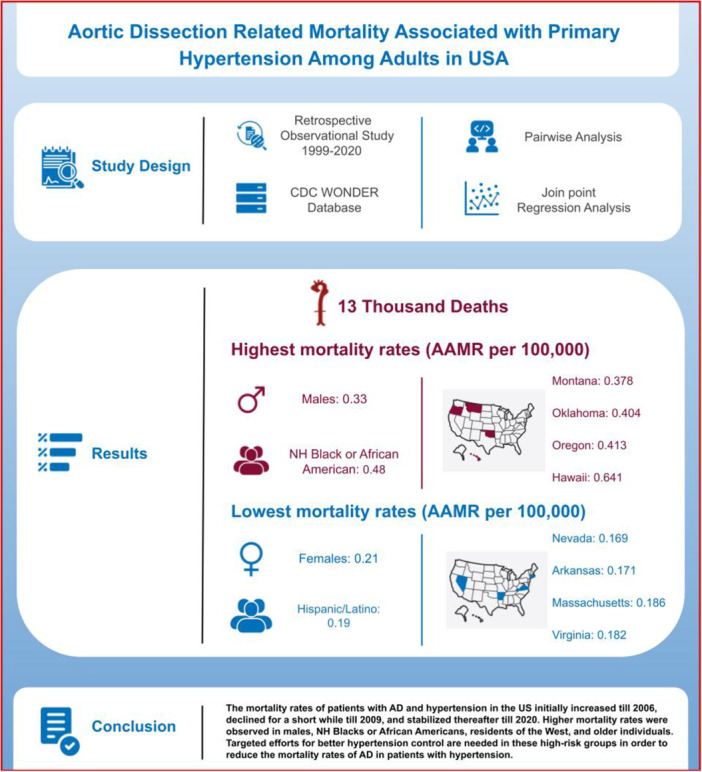

## 1 Introduction

Aortic dissection (AD) is a rare but life‐threatening condition involving a tear in the inner wall of the aorta, which can lead to severe complications such as aortic regurgitation, organ failure, cardiac tamponade, and rupture [1]. The global incidence of AD is estimated at 4.8 per 100 000 individuals/year, with significant variations across study designs and geographical regions [2]. In Oxfordshire, United Kingdom, the incidence was reported as 6 per 100 000 persons, whereas in Minnesota, United States (US), it was reported at an incidence of 3.5 per 100 000 persons [3]. AD is typically classified into two types: Type A (TAAD), which affects the ascending aorta, and Type B (TBAD), which begins distal to the left subclavian artery and spares the ascending aorta. Both types can present with severe chest or back pain, syncope, or even sudden cardiac death [4, 5, 6]. If left untreated, AD carries high mortality rates, highlighting the importance of early diagnosis and intervention. Key risk factors for AD include hypertension, atherosclerosis, and genetic connective tissue disorders like Marfan syndrome, as well as advanced age, male gender, and aortic aneurysms [7, 8]. Additionally, congenital abnormalities such as bicuspid or unicommissural aortic valves significantly increase the risk of certain types of dissection [7].

The prevalence of AD is expected to rise globally, driven by increasing rates of hypertension, particularly in aging populations [9]. Surgical repair remains the primary treatment, aiming to restore the function of the aorta [10]. Post‐surgical care often involves strict blood pressure management, as elevated blood pressure substantially increases the risk of reoperation and mortality. Studies indicate that maintaining a systolic blood pressure (SBP) of

The rise in hypertension and associated AD‐related mortality rates has become a pressing challenge worldwide. The aim of this study is to comprehensively analyse trends in mortality due to AD in adults with primary hypertension in the United States over the past two decades. The study will explore variations in mortality rates across different demographic groups, including age, gender, and racial disparities.

## 2 Methods

### 2.1 Study Design

We extracted data from the Centers for Disease Control and Prevention Wide‐Ranging Online Data for Epidemiologic Research (CDC WONDER) Database [11] to analyze trends in mortality due to AD in patients with hypertension aged 25 and above in the United States from 1999 to 2020. As a comparison cohort, all decedents with AD as the primary cause of mortality, regardless of contributing causes, aged 25 and above, were included from 1999 to 2020. Death certificate data is utilized by CDC WONDER to present both underlying and multiple causes of death, as well as demographic information.

We used the International Classification of Diseases, 10th Revision (ICD‐10) [12] codes I10 (essential [primary] hypertension) and I71.0 (dissection of aorta [any part]) to identify death certificates for individuals with AD as the underlying cause of death and hypertension as the multiple cause of death. Decedents with hypertension‐related cardiac and renal disease (I11−I14), secondary hypertension (I15), aortic aneurysm (I71.1–I71.9), or age < 25 years were excluded. Only records with complete demographic information were included in the analysis. Since this study utilized anonymized, publicly available data, it did not require ethical approval from an Institutional Review Board (IRB). This study conformed to the Strengthening the Reporting of Observational Studies in Epidemiology (STROBE) guidelines [13].

### 2.2 Data Abstraction

Data was stratified based on sex, race/ethnicity, census region, urbanization level, age group, and state. Sex was categorized as male or female. Race and ethnicity were divided into the following groups: Non‐Hispanic (NH) Asian or Pacific Islander, NH White, NH American Indian or Alaska Native, NH Black or African American, and Hispanic or Latino. Census regions were classified into four groups: Northeast, Midwest, South, and West. Urbanization levels were determined using the 2013 National Center for Health Statistics (NCHS) Urban‐Rural Classification Scheme for Counties [14]. Regions were grouped as either urban (large central metro, large fringe metro, medium metro, and small metro) or rural (micropolitan and noncore areas). Age was categorized into intervals of 10 years.

### 2.3 Statistical Analysis

Crude (CMR) and age‐adjusted mortality rates (AAMR) were calculated per 100 000 persons using annual population estimates provided by the US Census Bureau and embedded within the CDC WONDER database as denominators. Age adjustment was performed using the 2000 US standard population. Trends in mortality rates were analyzed using the Joinpoint Regression Program (Joinpoint version 5.1.0, National Cancer Institute), which uses serial permutation tests to examine repeated time trends and identify up to a single inflection point where the rate of change is significantly different [15]. For each time segment, the annual percentage change (APC) is obtained along with its corresponding 95% confidence interval (CI). APCs and average APCs (AAPCs) were reported with corresponding 95% CIs and *p* values, with statistical significance defined as a two‐sided *p*‐value < 0.05. We also performed a pairwise comparison with a comparison cohort of AD as the primary cause of death using the same program to examine whether there were statistically significant differences in APCs between both cohorts across various stratifications. A *p* value of <0.05 was considered to be significant.

## 3 Results

**Figure 1:**
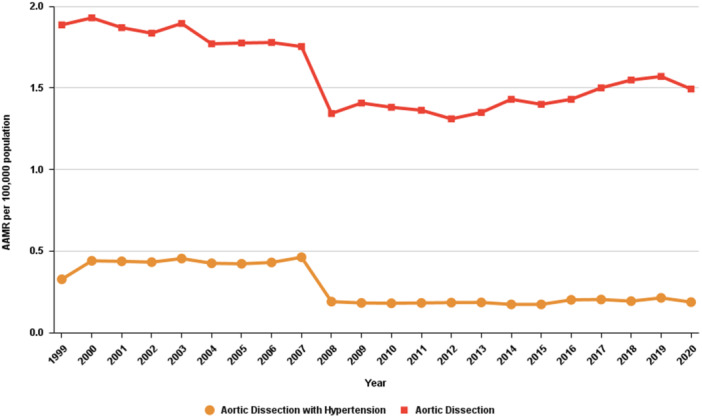
Trends in aortic dissection‐related mortality in patients with hypertension in the United States from 1999 to 2020.

During the study period, a total of 13 128 AD‐related deaths were reported in patients with primary HTN with an average AAMR of 0.275 (95% CI: 0.271 to 0.28) per 100 000 (Table 1). The cohort experienced a substantial increase in mortality from 1999 to 2006 (APC: 3.04*; 95% CI: 0.23 to 5.93), followed by a significant decline from 2006 to 2009 (APC: −27.60*; 95% CI: −43.02 to −8.02) and a stable trend from 2009 to 2020 (APC: 0.81; 95% CI: −1.03 to 2.69) (Table 2, Figure 1). The AD‐related mortality in people with primary HTN differs significantly when compared with AD alone (*p* = 0.0002, AAPC: −3.14; 95% CI: −6.34 to 0.16 and −1.02; 95% CI: −2.25 to 0.22 respectively) (Table 3).

**Table 1:** Demographic characteristics of deaths due to aortic dissection in patients with hypertension in the United States from 1999–2020.

Variable	Deaths	Population	AAMR (95% CI)
Overall	13 128	4 473 854 489	0.28 (0.27−0.28)
*Sex*			
Female	5773	2 319 297 578	0.21 (0.20−0.21)
Male	7355	2 154 556 911	0.33 (0.32−0.34)
*Age groups* a			
35 to 44 years	768	931 287 288	0.08 (0.08−0.09)
45 to 54 years	1832	927 576 220	0.20 (0.19−0.21)
55 to 64 years	2369	766 424 847	0.31 (0.30−0.32)
65 to 74 years	2662	510 458 341	0.52 (0.50−0.54)
75 to 84 years	3231	298 504 433	1.08 (1.05−1.12)
85+ years	2064	119 513 891	1.73 (1.65−1.80)
*Race/ethnicity*			
Hispanic or Latino	751	589 350 210	0.19 (0.18−0.21)
NH Asian or Pacific Islander	615	237 142 712	0.32 (0.30−0.35)
NH Black or African American	2340	518 937 524	0.48 (0.46−0.50)
NH White	9343	3 095 342 890	0.28 (0.27−0.28)
*Census region*			
Northeast	2263	827 193 779	0.25 (0.24−0.26)
Midwest	3085	969 567 311	0.30 (0.29−0.31)
South	4269	1 652 256 217	0.24 (0.23−0.25)
West	3511	1 024 837 182	0.36 (0.34−0.37)
*Urbanization*			
Urban	11 077	3 795 213 822	0.28 (0.27−0.28)
Rural	2051	678 634 169	0.26 (0.25−0.28)

Abbreviations: AAMR, age‐adjusted mortality rate; CI, confidence interval.

^a^ Crude mortality rate is used for analysis instead of age‐adjusted mortality rates for age groups.

**Table 2:** Annual percentage changes (APCs) and average annual percentage changes (AAPCs) in aortic dissection among patients with hypertension in the USA from 1999 to 2020.

Variable	Trend segment	Lower endpoint	Upper endpoint	APC (95% CI)	AAPC (95% CI)	*p* value
Entire cohort	1	1999	2006	3.04 (−0.39 to 9.36)	−3.14* (−4.14 to −1.95)	< 0.000001
	2	2006	2009	−27.60* (−33.05 to −15.86)		
	3	2009	2020	0.81 (−1.30 to 4.76)		
*Sex*						
Female	1	1999	2007	1.67 (−1.56 to 8.34)	−2.90* (−4.31 to −1.16)	0.006399
	2	2007	2010	−30.26* (−37.66 to −14.46)		
	3	2010	2020	3.36 (−0.62 to 13.65)		
Male	1	1999	2006	3.47 (−0.13 to 10.03)	−3.72* (−4.78 to −2.42)	< 0.000001
	2	2006	2009	−29.33 (−34.93 to −16.59)		
	3	2009	2020	0.07 (−2.31 to 4.33)		
*Age groups*						
35 to 44 years	1	1999	2006	6.07* (1.30 to 19.40)	0.39 (−1.16 to 2.37)	0.561088
	2	2006	2013	−10.62* (−25.80 to −5.62)		
	3	2013	2020	6.52* (0.45 to 25.57)		
45 to 54 years	1	1999	2006	5.45* (0.52 to 18.77)	−2.18 (−3.80 to 0.02)	0.052789
	2	2006	2009	−28.41* (−36.21 to −11.42)		
	3	2009	2020	1.54 (−2.03 to 11.87)		
55 to 64 years	1	1999	2006	3.24 (−0.30 to 9.32)	−2.81* (−3.76 to −1.65)	< 0.000001
	2	2006	2009	−29.19* (−34.27 to −17.70)		
	3	2009	2020	1.96 (−0.08 to 5.24)		
75 to 84 years	1	1999	2006	2.48 (−0.42 to 6.63)	−3.89* (−4.79 to −2.90)	< 0.000001
	2	2006	2009	−28.24* (−33.06 to −17.40)		
	3	2009	2020	−0.09 (−2.16 to 3.44)		
85+ years	1	1999	2007	3.94* (0.44 to 11.23)	−1.78 (−3.12 to 0.05)	0.055589
	2	2007	2010	−28.87* (−36.22 to −13.46)		
	3	2010	2020	3.41 (−0.24 to 11.36)		
*Race*						
Black or African American	1	1999	2006	6.58* (1.81 to 15.55)	−3.36* (−4.87 to −1.44)	0.001600
	2	2006	2009	−35.49* (−42.84 to −18.63)		
	3	2009	2020	1.39 (−2.16 to 9.08)		
White	1	1999	2007	−0.28 (−2.98 to 4.92)	−2.72* (−3.94 to −1.32)	0.001600
	2	2007	2010	−27.62* (−34.07 to −14.21)		
	3	2010	2020	4.22* (0.76 to 11.87)		
*Census region*						
Northeast	1	1999	2007	3.23* (0.55 to 7.70)	−3.58* (−4.85 to −2.27)	< 0.000001
	2	2007	2010	−29.52* (−35.82 to −17.12)		
	3	2010	2020	0.30 (−2.95 to 7.69)		
Midwest	1	1999	2006	2.06 (−1.27 to 8.47)	−2.66* (−3.57 to −1.51)	< 0.000001
	2	2006	2009	−25.19* (−30.41 to −13.43)		
	3	2009	2020	1.53 (−0.55 to 5.11)		
South	1	1999	2006	4.50* (0.73 to 10.73)	−2.40* (−3.42 to −1.16)	< 0.000001
	2	2006	2009	−31.64* (−37.25 to −18.76)		
	3	2009	2020	2.98* (0.60 to 7.06)		
West	1	1999	2006	3.23 (−0.42 to 10.40)	−3.39* (−4.43 to −2.06)	< 0.000001
	2	2006	2009	−27.20* (−32.95 to −14.64)		
	3	2009	2020	0.06 (−2.29 to 4.44)		
*Urbanization*						
Urban	1	1999	2006	2.45 (−1.01 to 8.78)	−3.56* (−4.55 to −2.33)	< 0.000001
	2	2006	2009	−27.46* (−32.99 to −15.23)		
	3	2009	2020	0.29 (−1.99 to 4.43)		
Rural	1	1999	2007	2.58 (−1.34 to 10.37)	−2.04* (−3.52 to −0.08)	0.043591
	2	2007	2010	−30.58* (−38.84 to −14.17)		
	3	2010	2020	4.70* (0.42 to 15.75)		

Abbreviations: AAPC, average annual percent change; APC, annual percent change; CI, confidence interval.

* Indicates that the APC or AAPC is significantly different from zero at the alpha = 0.05 level.

**Table 3:** Pairwise comparison between aortic dissection‐related mortality rates in patients with hypertension and overall aortic dissection‐related mortality rates in the USA from 1999 to 2020.

Variable	Aortic dissection‐related mortality rates in patients with hypertension		Overall aortic dissection‐related mortality rates		*p* value for AAPC comparison
	AAPC (95% CI)	*p* value	AAPC (95% CI)	*p* value	
Overall	−3.14 (−6.34 to 0.16)	0.062108	−1.02 (−2.25 to 0.23)	0.108542	0.000222*
*Sex*					
Male	−3.72 (−7.57 to 0.30)	0.069006	−1.30 (−2.87 to 0.29)	0.108620	0.000222*
Female	−2.90 (−7.24 to 1.63)	0.205726	−1.19 (−2.64 to 0.27)	0.110401	0.000222*
*Age groups*					
35 to 44 years	0.33 (−4.32 to 5.21)	0.892150	1.28* (0.70 to 1.87)	0.000174	0.000222*
45 to 54 years	−2.18 (−7.24 to 3.15)	0.415815	0.56 (−1.58 to 2.75)	0.611586	0.000222*
55 to 64 years	−2.81 (−7.22 to 1.82)	0.229945	−1.16* (−2.26 to −0.05)	0.041347	0.000222*
65 to 74 years	−3.49 (−9.99 to 3.47)	0.317399	−2.32* (−3.77 to −0.84)	0.002183	0.000222*
75 to 84 years	−3.89 (−8.25 to 0.66)	0.092894	−2.30* (−3.64 to −0.96)	0.000862	0.000222*
85+ years	−1.78 (−7.73 to 4.55)	0.572392	−0.24 (−3.06 to 2.67)	0.871830	0.000222*
*Race*					
Hispanic or Latino	−4.36 (−16.05 to 8.96)	0.502655	−1.50 (−4.52 to 1.61)	0.340180	0.000222*
NH White	−2.72 (−6.35 to 1.05)	0.155127	−1.00 (−2.02 to 0.03)	0.058092	0.000222*
NH Black or African American	−3.36 (−9.32 to 3.00)	0.293666	−0.10 (−2.48 to 2.35)	0.937988	0.000222*
NH Asian or Pacific Islander	−8.25* (−9.79 to −6.68)	< 0.000001	−3.26* (−4.98 to −1.51)	0.000302	0.000444*
*Census region*					
Northeast	−3.58 (−7.10 to 0.08)	0.054811	−1.14 (−2.63 to 0.37)	0.136896	0.000222*
Midwest	−2.64 (−7.09 to 2.03)	0.263494	−0.80 (−1.65 to 0.05)	0.064322	0.000444*
South	−2.40 (−7.78 to 3.30)	0.401363	−0.69 (−2.03 to 0.67)	0.317430	0.000222*
West	−3.39 (−7.86 to 1.31)	0.154477	−1.49* (−2.59 to −0.38)	0.008634	0.000222*
*Urbanization*					
Urban	−3.56 (−7.80 to 0.88)	0.114154	−1.11 (−2.48 to 0.27)	0.114793	0.000222*
Rural	−2.04 (−10.56 to 7.31)	0.658147	−0.38 (−1.96 to 1.24)	0.645423	0.000222*

### 3.1 Trends by Sex

**Figure 2:**
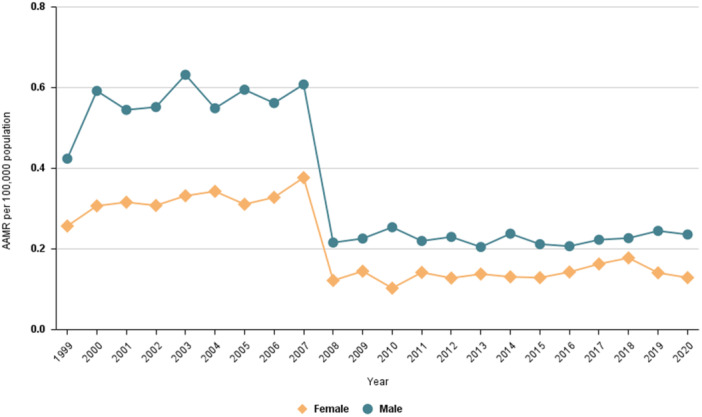
Trends in aortic dissection‐related mortality in patients with hypertension stratified by gender in the United States from 1999 to 2020.

Males with primary HTN had a higher AD‐related mortality rate compared to females (AAMR: 0.328; 95% CI: 0.32 to 0.336 and AAMR: 0.208; 95% CI: 0.203 to 0.214, respectively) (Table 1). Males experienced a substantial increase in mortality from 1999 to 2006 (APC: 3.47*; 95% CI: 0.24 to 6.69), followed by a significant decline from 2006 to 2009 (APC: −29.33*; 95% CI: −47.33 to −11.32). The AAMR remained stable from 2009 to 2020 (APC: 0.07; 95% CI: −1.99 to 2.13). A similar trend was observed in females, with stable AAMR from 1999 to 2007 (APC: 1.67; 95% CI: −0.90 to 4.23), followed by a significant decrease from 2007 to 2010 (APC: −30.26*; 95% CI: −50.04 to −10.48). In contrast to males, AAMR in females showed a significant increase after 2010 (APC: 3.36*; 95% CI: 0.89 to 5.83) (Table 2, Figure 2).

The AD‐related mortality in people with primary HTN differed significantly from that of AD alone in both males (*p* = 0.0002; AAPC: −3.72; 95% CI: −7.57 to 0.30 for AD with primary HTN and AAPC: −1.30; 95% CI: −2.87 to 0.29 for AD alone) and females (*p* = 0.0002; AAPC: −2.90 95% CI: −7.24 to 1.63 for AD with primary HTN and AAPC: −1.19; 95% CI: −2.64 to 0.27 for AD alone) (Table 3).

### 3.2 Trends by Race

When stratified by racial groups, NH Black or African American individuals had the highest mortality rate (AAMR: 0.48, 95% CI: 0.46 to 0.50), followed by NH Asians or Pacific Islanders (AAMR: 0.32, 95% CI: 0.30 to 0.35) and NH Whites (AAMR: 0.28, 95% CI: 0.27 to 0.28). Hispanics exhibited an AAMR of 0.19 (95% CI: 0.18 to 0.21), while NH American Indian or Alaska Native individuals had the lowest AAMR of 0.17 (95% CI: 0.12 to 0.23) (Table 1).

For NH Black or African American individuals, the cohort experienced a substantial increase in mortality from 1999 to 2006 (APC: 6.58*; 95% CI: 1.63 to 11.76), followed by stable trends from 2006 to 2009 (APC: −35.49; 95% CI: −59.30 to 2.24) and 2009 to 2020 (APC: 1.39; 95% CI: −1.83 to 4.72). Asians or Pacific Islanders experienced a significant decline in mortality from 2000 to 2020 (AAPC: −8.25*; 95% CI: −9.79 to −6.68). NH Whites cohort experienced a stable trend in mortality from 1999 to 2007 (APC: −0.28; 95% CI: −2.30 to 1.77), followed by a significant decline from 2007 to 2010 (APC: −27.62*; 95% CI: −45.21 to −4.36). In contrast, there was a substantial increase in mortality from 2010 to 2020 (APC: 4.22*; 95% CI: 2.21 to 6.26). Hispanics or Latinos experienced stable mortality rates from 1999 to 2007 (APC: 2.66; 95% CI: −3.10 to 8.76), from 2007 to 2011 (APC: −26.57; 95% CI: −64.76 to 53.01), and from 2011 to 2020 (APC: 0.99; 95% CI: −4.70 to 7.03) (Table 2, Supporting Information S1: Figure 1). The AD‐related mortality in people with primary HTN differed significantly from that of AD alone in NH Blacks (*p* = 0.0002), NH Asians (*p* = 0.0004), NH Whites (*p* = 0.0002) and Hispanics or Latinos (*p* = 0.0002) (Table 3).

### 3.3 Trends by Census Region

When stratified by region, the highest mortality rate was observed in the West (AAMR: 0.36, 95% CI: 0.34 to 0.37), followed by the Midwest (AAMR: 0.30, 95% CI: 0.29 to 0.31), the Northeast (AAMR: 0.25, 95% CI: 0.24 to 0.26), and the South (AAMR: 0.24, 95% CI: 0.23 to 0.25) (Table 1). For most regions, trends in mortality rates followed a similar pattern. From 1999 to 2006, mortality rates remained stable in the West (APC: 3.23; 95% CI: −0.73 to 7.36) and the Midwest (APC: 2.06; 95% CI: −1.73 to 6.00), while the South experienced a substantial rise (APC: 4.50*; 95% CI: 0.02 to 9.17). This was followed by a stable trend in mortality rates from 2006 to 2009 across the West (APC: −27.20; 95% CI: −48.12 to 2.16), the Midwest (APC: −25.19; 95% CI: −46.52 to 4.64), and the South (APC: −31.64; 95% CI: −54.56 to 2.85). From 2009 to 2020, the trends varied again: the West showed stability in death rates (APC: 0.06; 95% CI: −2.46 to 2.64), the Midwest saw also showed a stable trend (APC: 1.53; 95% CI: −0.94 to 4.07), and the South experienced a moderate rise (APC: 2.98*; 95% CI: 0.00 to 6.04). The Northeast experienced a substantial increase in mortality from 1999 to 2007 (APC: 3.23*; 95% CI: 1.16 to 5.34), followed by a significant decline from 2007 to 2010 (APC: −29.52*; 95% CI: −46.23 to −7.62). There was a stability in death rates from 2010 to 2020 (APC: 0.30; 95% CI: −1.80 to 2.44) (Table 2, Supporting Information S1: Figure 2). The AD‐related mortality in people with primary HTN differed significantly from that of AD alone in Midwest (*p* = 0.0004), South (*p* = 0.0002), West (*p* = 0.0002) and Northeast (*p* = 0.0002) (Table 3).

### 3.4 Trends by Urbanization

Most deaths occurred in urban areas, with an AAMR of 0.28 (95% CI: 0.27 to 0.28), compared to rural areas, which had a slightly lower AAMR of 0.26 (95% CI: 0.25 to 0.28) (Table 1). The trends in mortality rates for both urban and rural areas followed a similar pattern. From 1999 to 2006, mortality rates remained stable in both settings: APC: 2.45; 95% CI: −1.05 to 6.07 for urban, while APC: 2.58; 95% CI: −2.53 to 7.95 for rural areas. This was followed by another period of stability in both regions from 2006 to 2009 (urban areas: APC: −27.46; 95% CI: −47.55 to 0.33, and rural areas: APC: −30.58; 95% CI: −64.29 to 34.92). From 2009 to 2020, mortality rates did not change significantly in urban areas (APC: 0.29; 95% CI: −1.97 to 2.61), as well as in rural areas (APC: 4.70; 95% CI: −0.31 to 9.96) (Table 2, Supporting Information S1: Figure 3). The AD‐related mortality in people with primary HTN differed significantly from that of AD alone in both Urban (*p* = 0.0002; AAPC: −3.56; 95% CI: −7.80 to 0.87 for AD with primary HTN and AAPC: −1.11; 95% CI: −2.48 to 0.27 for AD alone) and Rural (*p* = 0.0002; AAPC: −2.03; 95% CI: −10.56 to 7.30 for AD with primary HTN and AAPC: −0.38; 95% CI: −1.96 to 1.24 for AD alone) (Table 3).

### 3.5 Trends by Age Group

Mortality rates varied significantly across age groups. The lowest mortality rate was observed in the 25–34 year age group (CMR: 0.02, 95% CI: 0.02 to 0.03). Mortality rates gradually increased with age, with the 35–44 year group having an CMR of 0.08 (95% CI: 0.08 to 0.09), and the 45–54 year group having an CMR of 0.20 (95% CI: 0.19 to 0.21). The 55‐64 year group exhibited an CMR of 0.31 (95% CI: 0.30 to 0.32), while the 65‐74 year group had an CMR of 0.52 (95% CI: 0.50 to 0.54). The highest mortality rates were observed in the 75‐84 year group (CMR: 1.08, 95% CI: 1.05 to 1.12), and the 85 and above group had the highest CMR of 1.73 (95% CI: 1.65 to 1.80) (Supporting Information S1: Figure 4). The AD‐related mortality in people with primary HTN differed significantly from that of AD alone in all age groups (*p* = 0.0002) (Table 3).

### 3.6 Trends by States

Differences in AAMRs were notable across states. The states with the highest AAMRs were Hawaii (AAMR = 0.64; 95% CI = 0.54 to 0.75), Oregon (AAMR = 0.41; 95% CI = 0.36 to 0.47), and Oklahoma (AAMR = 0.40; 95% CI = 0.35 to 0.46). These states exhibited rates that were several times higher compared to the states with the lowest AAMRs, such as Virginia (AAMR = 0.18; 95% CI = 0.16 to 0.21), Arkansas (AAMR = 0.17; 95% CI = 0.13 to 0.22), and Nevada (AAMR = 0.17; 95% CI = 0.13 to 0.22) (Supporting Information S1: Figure 5).

## 4 Discussion

We uncovered several key findings in our study regarding the trends and disparities in mortality related to AD in patients with primary hypertension. The mortality rates initially increased till 2006, then declined for a short while till 2009 before stabilizing. Males had higher mortality rates than females, but the mortality rates were initially stable in females till 2007 and increased after 2010. NH Blacks or African Americans showed the highest mortality rates while NH American Indian or Alaska Natives showed the lowest. The West showed the highest mortality rates while the South showed the lowest. Urban areas had very slightly higher mortality rates than rural areas. The younger age groups showed lower mortality rates. Lastly, the mortality rates for AD with primary HTN were significantly different from the mortality rates for AD alone in the overall population, in both sexes, across all races, across all regions, in both urban and rural settings, and in all age groups.

The mortality trends observed in our study stand in stark contrast to those observed in patients with AD alone. A previously conducted study in the US revealed an initial decline in mortality rates, followed by increasing mortality after 2012 due to AD alone [16]. Rethy et al. showed a general increase in deaths from hypertension over the last two decades in the US, but a period of declining mortality was observed from 2003 to 2012 and the deaths in patients with hypertension and ischemic heart disease decreased throughout the period [17]. The rates of hypertension control also increased from 1999 to 2010 in the US, with a plateaued rate thereafter till 2014 and declining rates from 2015 to 2018 [18]. As hypertension is a major risk factor for the incidence of AD in the first place [19], the decline in trends for AD‐related mortality in patients with hypertension from 2006 to 2009 and the stable rates thereafter may be attributed to better blood pressure control and patient education in recent years. Our study also showed a significant difference between mortality rates of patients with both AD and hypertension compared to AD alone. Previous studies have demonstrated a positive association of both systolic and diastolic blood pressure with the risk of death in AD [20]. Furthermore, hypertension leads to poorer surgical outcomes and confers a worse prognosis after surgery for TAAD [21] and is an independent risk factor for early mortality in patients with acute TBAD [22]. However, the stable trends of mortality in patients with AD and hypertension despite the increasing rates for AD alone suggest that the contribution of other causes might be increasing.

Our study showed higher mortality rates in males than in females. This is consistent with the mortality rates for AD alone in the US [16]. Previous studies suggest a higher incidence of AD with a declining trend in men, but no decrease in women [23]. The greater incidence of AD explains the greater mortality rates in males. On the other hand, women with AD generally present late and are more commonly hypertensive [23]. The rising rates of uncontrolled hypertension in older patients in the US in coupled with a greater prevalence of uncontrolled hypertension in older women who are more prone to AD likely underlie the increasing mortality rates for females with AD and hypertension in recent years [18, 24].

NH African Americans have suffered a higher mortality due to AD compared to other races in recent years [16]. They have also carried an almost disproportionately greater burden of disease of hypertension in the country [25]. These conclusions are consistent with our results of higher mortality in the NH African American population. It has been observed that NH African Americans develop hypertension not only earlier but also with a greater severity, worse control rates, and have a higher risk of cardiovascular complications despite high disease awareness [26, 27, 28, 29, 30, 31]. Additionally, African Americans at the time of surgical repair of AD are younger and have worse control of hypertension [32, 33, 34, 35]. The same studies suggest that African Americans have higher rates of readmission and re‐intervention [32, 33, 34, 35, 36, 37, 38], and while most suggest that post‐operative mortality for African Americans is comparable to other races, some do report higher post‐ op mortality in Blacks [37]. Moreover, some studies pinpoint that the African Americans presenting with AD are more likely to belong to a lower socioeconomic status [34, 35, 38] which implies that despite the recent efforts to implement healthcare equity across all ethnicities, much work still needs to be done.

The West region has the highest mortality overall due to AD in individuals with hypertension, while the South has the lowest. This finding overlaps with some, but not all the states reported in prior literature to have higher same cause mortality [25, 29]. Additionally, we found that mortality rates in urban areas were very slightly higher than rural areas, similar to various other reports by prior research [16]. Lower socioeconomic status is known to be associated with higher mortality rates in AD [39, 40]. This could explain the higher rural mortality, especially when considering coexistent difficulties in healthcare access for the rural population at large. However, our finding regarding the high mortality in the West is interesting, since the region has relatively lower prevalence of hypertension [41]. Furthermore, these areas lack many other risk factors for AD and hypertension discussed beforehand. We hypothesize that the high mortality could be due to an interplay of underreporting in the lower socioeconomic regions such as the South, and unrecognized or understudied risk factors in the West.

The mortality rates due to AD in patients with hypertension were higher in older age groups. This is consistent with data for mortality due to AD alone in the US [16]. A report from the International Registry of Acute AD suggests that lower rates of surgical aortic repair and greater surgical mortality with age; age above 70 years was considered an independent risk factor for mortality [42, 43]. A single‐center study in the United States by Nithikasem et al. revealed a greater delay in time to surgery for patients above 70 years with AD [44]. Hypertension control has been declining in adults above 40 years of age in the United States, further compounding the effect of age in our study [18]. Elderly patients also frequently present with other comorbidities such as atherosclerosis and diabetes mellitus, which may contribute to poor survival rates and great mortality [7].

To our knowledge, this is the first nationwide study exploring the trends and disparities in mortality due to AD in patients with hypertension. The main strength of our study is the robust sample size, which allows delineation of temporal trends and disparities in the entire US population for the past two decades. This study has several limitations inherent to the use of death certificate–based administrative data. First, misclassification or underreporting of hypertension and AD on death certificates may have led to underestimation of true mortality [45]. Second, the database does not allow differentiation between Stanford type A and type B AD, nor does it capture blood pressure control, treatment strategies, surgical intervention, comorbid conditions, or socioeconomic status. Additionally, causal inferences cannot be made due to the observational design. Third, we could not assess the impact of other comorbidities or socioeconomic status. Lastly, it is pertinent to mention that the mortality rates used are for the general population, which allow better comparison with existing literature, instead of proportional mortality rates. Despite these limitations, the use of population‐based mortality rates allows meaningful national trend comparisons over time.

We observed several disparities in the mortality rates due to AD and hypertension. Better control of hypertension in the general population, providing timely interventions for the elderly, and equitable provision of healthcare are crucial to reduce the mortality rates. Patient education regarding hypertension and the risk of fatal AD is critical, especially in the elderly. Future studies should focus on the impact of other comorbidities on AD and assess the disparities separately in TAAD and TBAD. Further research is needed to identify factors which manifest as high mortality in the West as well as exploration of lower mortality in the South.

## 5 Conclusion

The mortality rates of patients with AD and hypertension in the US initially increased till 2006, declined for a short while till 2009, and stabilized thereafter till 2020. Higher mortality rates were observed in males, NH Blacks or African Americans, residents of the West, and older individuals. Targeted efforts for better hypertension control are needed in these high‐risk groups in order to reduce the mortality rates of AD in patients with hypertension.

## Author Contributions

**Shahzaib Ahmed:** conceptualization, project administration, formal analysis, writing – original draft, writing – review and editing. **Zain Ali Nadeem:** validation, writing–original draft, writing – review and editing. **Aimen Nadeem:** validation, writing – original draft, writing – review and editing. **Hamza Ashraf:** validation, writing – original draft, writing – review and editing. **Umar Akram:** validation, writing – original draft, writing – review and editing. **Eeman Ahmad:** validation, writing – original draft, writing – review and editing. **Shoaib Ahmad:** validation, writing – review and editing; **Ibrahim Nagmeldin Hassan:** validation, writing – original draft, writing – review and editing. **Irfan Ullah:** validation, writing – review, and editing. **Raheel Ahmed:** validation, writing – review, and editing. **Anwar A. Chahal:** validation, writing – review, and editing. **Rui Bebiano Da Providencia E. Costa:** supervision, writing – review and editing. **Chadi Alraies:** supervision, writing – review and editing.

## Funding

The authors received no specific funding for this work.

## Conflicts of Interest

The authors declare no conflicts of interest.

## Supporting information


**Supporting Figure 1:** Trends in Aortic Dissection‐Related Mortality in Patients with hypertension stratified by race/ethnicity in the United States from 1999 to 2020. **Supporting Figure 2:** Trends in Aortic Dissection‐Related Mortality in Patients with hypertension stratified by census region in the United States from 1999 to 2020. **Supporting Figure 3:** Trends in Aortic Dissection‐Related Mortality in Patients with hypertension stratified by urbanisation in the United States from 1999 to 2020. **Supporting Figure 4:** Trends in Aortic Dissection‐Related Mortality in Patients with hypertension stratified by ten‐year age groups in the United States from 1999 to 2020. **Supporting Figure 5:** State‐wise deaths and Age Adjusted Mortality rates (AAMRs) in Aortic Dissection‐Related Mortality in Patients with hypertension in the United States from 1999 to 2020.

## Data Availability

All data generated or analyzed during this study are included in this published article and its Supporting Information S1: Files and are freely available on the CDC WONDER database.
