# Supplementary material for: Mortality Due to Aortic Dissection in Adults With Primary Hypertension: A Nationwide Analysis Over Two Decades
Source: Clin Cardiol. 2026 Feb 5;49(2):e70269. doi: 10.1002/clc.70269 (PMC12877423; doi:10.1002/clc.70269)

**Supplementary File

Mortality due to aortic dissection in adults with primary hypertension:**

**A nationwide analysis over two decades**

This file is created by the authors to provide a better understanding of their work.

**Supplementary Figure 1:** Trends in Aortic Dissection-Related Mortality in Patients with hypertension stratified by race/ethnicity in the United States from 1999 to 2020


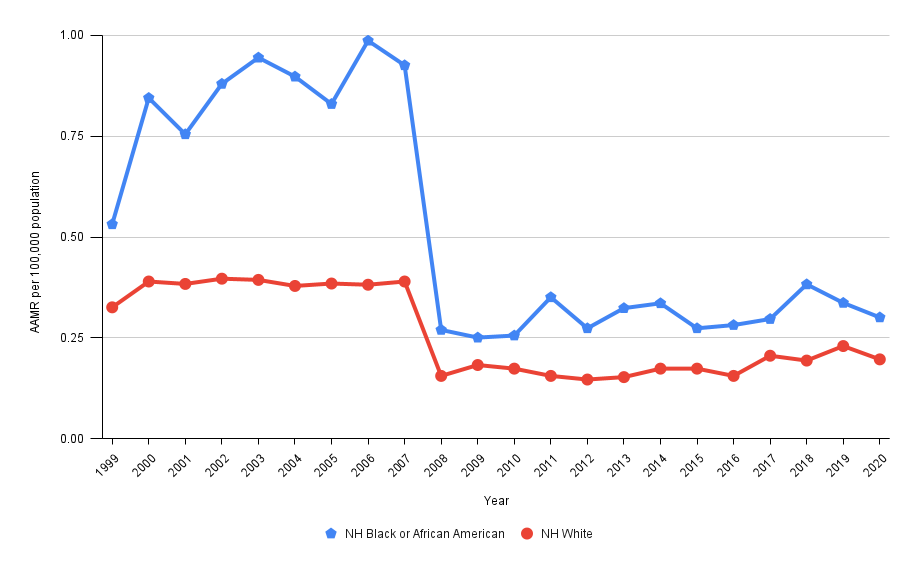


**Supplementary Figure 2:** Trends in Aortic Dissection-Related Mortality in Patients with hypertension stratified by census region in the United States from 1999 to 2020


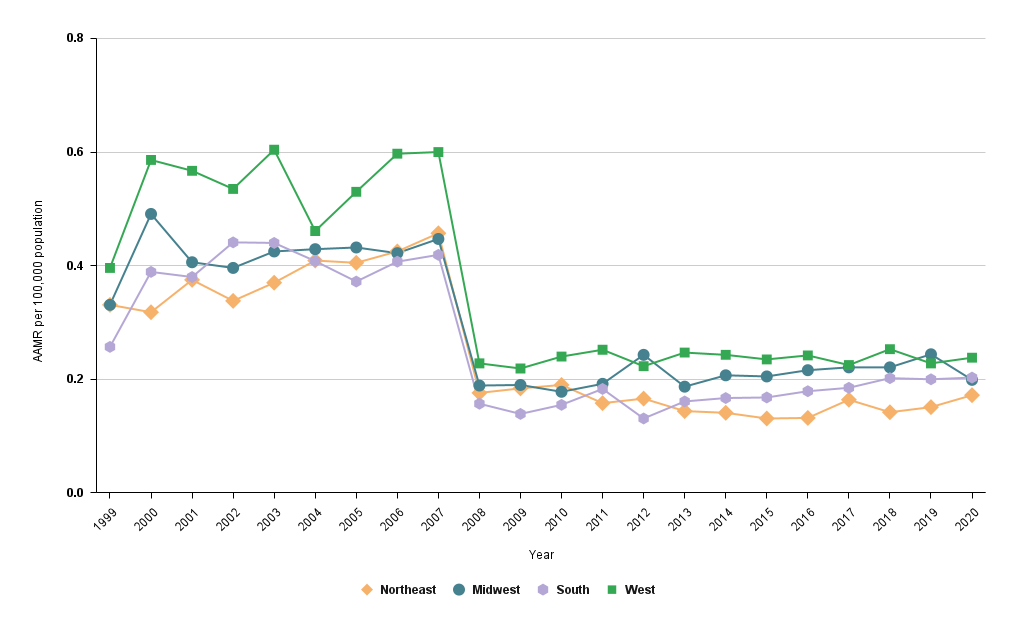


**Supplementary Figure 3:** Trends in Aortic Dissection-Related Mortality in Patients with hypertension stratified by urbanisation in the United States from 1999 to 2020


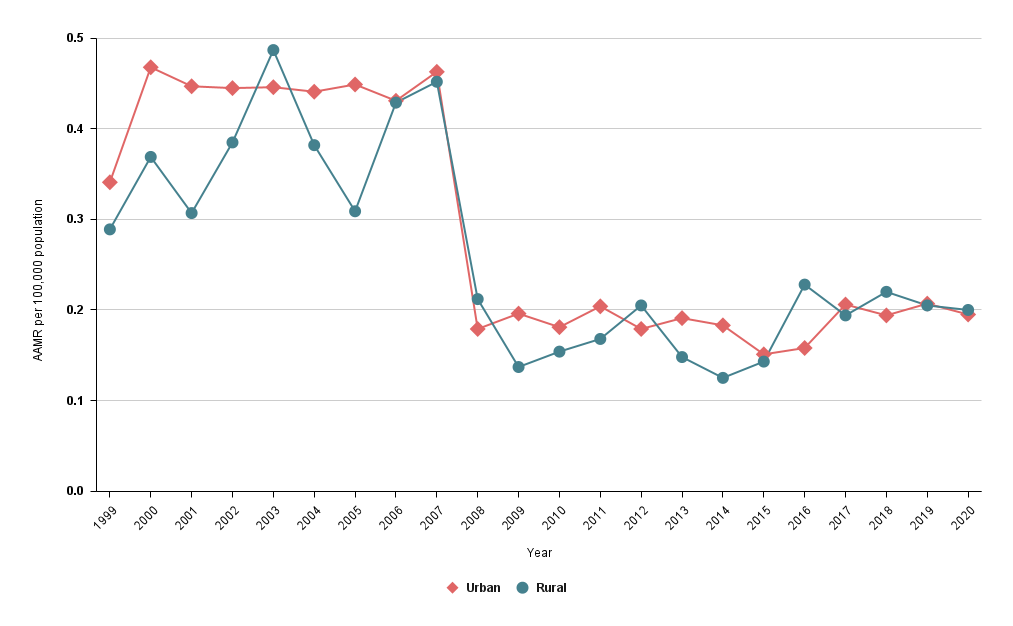


**Supplementary Figure 4:** Trends in Aortic Dissection-Related Mortality in Patients with hypertension stratified by ten-year age groups in the United States from 1999 to 2020


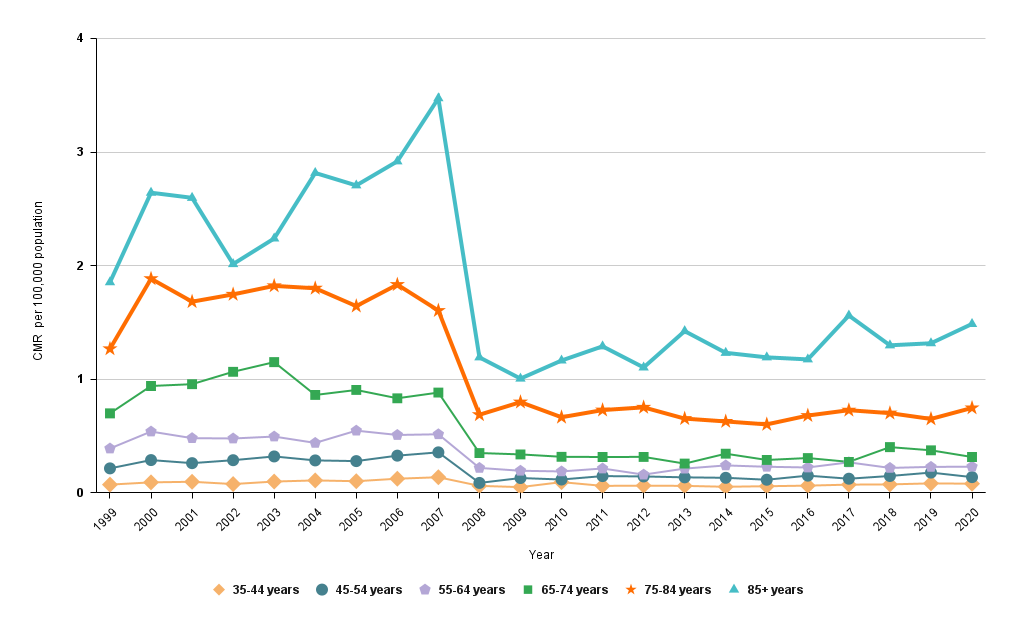


**Supplementary Figure 5:** State-wise deaths and Age Adjusted Mortality rates (AAMRs) in Aortic Dissection-Related Mortality in Patients with hypertension in the United States from 1999 to 2020


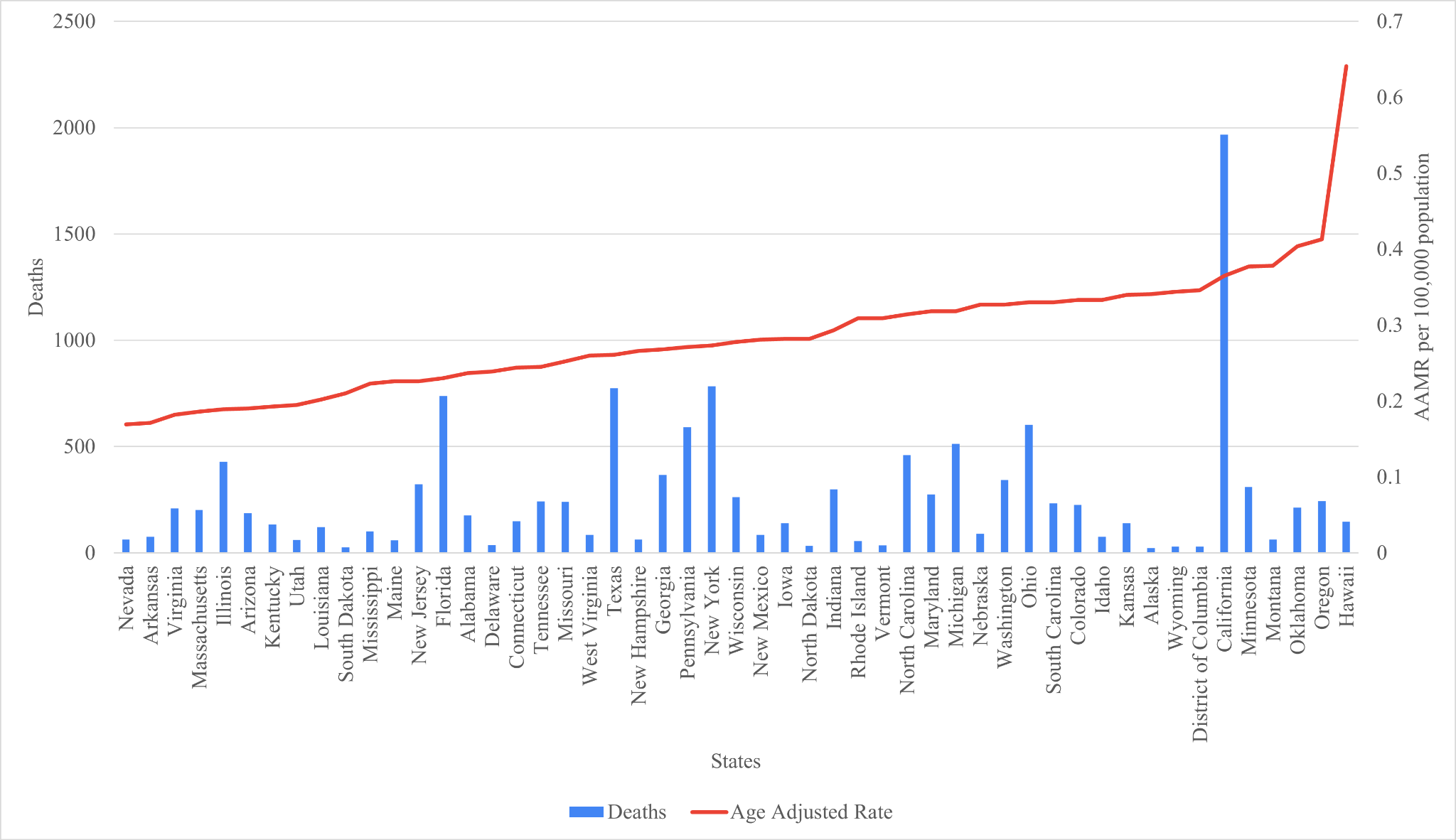

Supplement: Supplementary file 1 — Supporting Figure 1: Trends in Aortic Dissection‐Related Mortality in Patients with hypertension stratified by race/ethnicity in the United States from 1999 to 2020. Supporting Figure 2: Trends in Aortic Dissection‐Related Mortality in Patients with hypertension stratified by census region in the United States from 1999 to 2020. Supporting Figure 3: Trends in Aortic Dissection‐Related Mortality in Patients with hypertension stratified by urbanisation in the United States from 1999 to 2020. Supporting Figure 4: Trends in Aortic Dissection‐Related Mortality in Patients with hypertension stratified by ten‐year age groups in the United States from 1999 to 2020. Supporting Figure 5: State‐wise deaths and Age Adjusted Mortality rates (AAMRs) in Aortic Dissection‐Related Mortality in Patients with hypertension in the United States from 1999 to 2020. [file CLC-49-e70269-s001.docx]
